# Interposed Abdominal Compression CPR for an Out-of-Hospital Cardiac Arrest Victim Failing Traditional CPR

**DOI:** 10.5811/westjem.2015.6.26082

**Published:** 2015-10-20

**Authors:** Christian D. McClung, Alexander J. Anshus

**Affiliations:** * Keck School of Medicine of the University of Southern California, Department of Emergency Medicine, Department of Molecular Microbiology and Immunology, Los Angeles, California; † University of California, Irvine, School of Medicine, Irvine, California

## Abstract

Interposed abdominal compression cardiopulmonary resuscitation (IAC-CPR) is an alternative technique to traditional cardiopulmonary resuscitation (CPR) that can improve perfusion and lead to restoration of circulation in patients with chest wall deformity either acquired through vigorous CPR or co-morbidity such as chronic obstructive pulmonary disease. We report a case of out-of-hospital cardiac arrest where IAC-CPR allowed for restoration of spontaneous circulation and eventual full neurologic recovery when traditional CPR was failing to generate adequate pulses with chest compression alone.

## CASE REPORT

While chest compression cardiopulmonary resuscitation (CPR) is the primary resuscitation technique, it is not the only technique that can be applied, and in some cases it may fail to provide adequate perfusion pressure to restore circulation. We report on the use of interposed abdominal compression CPR (IAC-CPR) in an out-of-hospital cardiac arrest victim.

A 79-year-old female with history of chronic obstructive pulmonary disease (COPD), chronic renal failure, dialysis dependence, diabetes mellitus, and hypertension who was being treated for a respiratory infection with Levaquin for one day, awoke in the early morning with an ill feeling. A few minutes later she had a witnessed cardiac arrest, emergency medical services (EMS) was immediately notified, and bystander CPR was initiated. Upon paramedic arrival, she had an agonal rhythm and was pulseless. Paramedics initiated advanced cardiac life support, while standard CPR was continued. She was intubated, given two rounds of epinephrine, and transported to the emergency department (ED). Upon arrival after more than 20 minutes from her arrest, her rhythm was asystole, she remained apneic, and pupils were equal and reactive. The position of her endotracheal tube, placed in the pre-hospital setting, was confirmed and an orogastric tube was passed, with suction applied. She had a right anterior-chest dialysis catheter, which was used to deliver two ampules of 8.4% NaHCO_3_, two ampules of CaCl, and 40 units of vasopressin. There were no interruptions of the chest compressions from EMS handoff or during procedures. During the secondary assessment it was noted that the pulsations with chest compression were nearly undetectable despite what appeared to be adequate compression depth and rate. The intermittent pulse checks that occurred over the ensuing nine minutes were with pulseless electrical activity. The physician took over chest compressions and noted the severe chest wall deformity and lack of chest-recoil. Following that discovery, we initiated IAC-CPR, and her pulses with chest compression significantly improved. The rate of abdominal compression was matched 1:1 to chest compressions at about 100 chest compressions per minute. Her rhythm changed to ventricular fibrillation, and she underwent defibrillation with 200J (biphasic), with restoration of spontaneous circulation at 16 minutes after ED arrival. Her neurologic assessment remained unresponsive but with equal reactive pupils. Her electrocardiogram was interpreted as sinus rhythm with a ventricular rate of 56, right bundle branch block, without any ST-segment elevations. Chest radiograph demonstrated good position of supportive devices and bilateral lower lobe infiltrates were noted.

Initial laboratory studies revealed normal potassium, elevated troponin I (6.63), creatinine 4.2, and arterial blood gas demonstrated pH 7.24, pCO_2_ 42, pO_2_ 111, HCO_3_ 18, 94% saturation, and base excess of -9. A bedside limited echocardiography revealed global hypokinesis and absence of a pericardial effusion. She was hypotensive, treated with norepinephrine, and placed on a continuous bicarbonate infusion. Empiric intravenous antibiotics were given.

She was admitted to the intensive care unit. Later that morning, she underwent stenting of a subtotal occlusion of the proximal left anterior descending artery. The following day the patient was responsive and was extubated on hospital day two, with complete neurologic recovery with complaints of chest discomfort but no abdominal pain.

## DISCUSSION

Most emergency physicians are unfamiliar with IAC-CPR even though it was described in previous editions of Roberts and Hedges Clinical Procedures in Emergency Medicine. In this case, standard chest compression CPR alone was not effective, and without changing our strategy the patient would not have likely achieved adequate perfusion to allow restoration of spontaneous circulation. Viewing the chest wall integrity as dynamic and changing during resuscitation efforts, assessment of the adequacy of pulses and other markers of perfusion were key to determining that alternative resuscitation strategies were necessary.

This technique requires three providers, one to provide bag-valve mask respirations, one for the chest wall and another for the abdomen. The synchronization of the abdominal compressions is challenging but merely requires a counter pulsation for every chest compression with a slightly caudal and deep compression at the mid-abdomen 5cm above the umbilicus. The International Liaison Committee on Resuscitation (ILCOR) supports the use of IAC-CPR in hospital when sufficiently trained personnel are available.^1^ Our case differs from published recommendations since there are no studies of using this technique as a rescue strategy from failed traditional methods.

There are many possible mechanisms that may explain the efficacy of IAC-CPR. Sack et al.^2^ offered two of these mechanisms. The first postulates that by compressing the abdominal aorta, the aortic diastolic blood pressure is improved, and this may lead to retrograde flow to the coronary arteries and brain. The second involves “priming of the thoracic pump,” which states that global increases in intrathoracic pressure are equally transmitted to the heart, lung, and pulmonary vessels, such that the intrathoracic blood pool is advanced with each compression.^3^ Lastly, by compressing the abdomen, we are likely increasing venous return and improving stroke volumes.^4^ Ultimately, this technique is aimed at improving coronary perfusion pressure (CPP), which has been demonstrated to be essential to establish return of spontaneous circulation (ROSC) when either maximal CPP>15mmHg or peak diastolic CPP>12mmHg is achieved.^5^–^7^

IAC-CPR has been shown in animal models and human clinical studies to increase several hemodynamic factors and improve survival outcomes. In canine and porcine models, it has been shown to increase cerebral perfusion,^8^–^9^ cardiac output,^8^,^10^ carotid artery perfusion,^11^–^12^ coronary artery perfusion pressure,^11^,^13^ systolic and diastolic arterial pressure,^10^,^13^ and oxygen delivery.^8^ In human studies, it has been demonstrated to increase cardiac output,^4^,^14^–^15^ systolic and diastolic arterial pressures,^14^–^15^ and to improve clinical outcomes such as ROSC,^2^,^16^–^17^ 24-hour survival,^2^,^16^ survival to discharge,^2^,^17^ and six-month survival.^17^ The studies demonstrating improved clinical outcomes involved in-hospital cardiac arrest, in contrast to outcomes involved with out-of-hospital cardiac arrest, which were not significantly changed by this method. McDonald^18^ found no increase in systolic arterial pressure, and Mateer et al.,^19^ found no change in survival outcome. Sack^20^ postulated that this may be due to decreased vascular tone associated with prolonged cardiac arrest.

A previously stated concern with the addition of abdominal compressions during CPR is abdominal organ injury. IAC-CPR has been shown through several clinical studies not to cause any major abdominal injury.^2^,^4^,^14^–^17^,^19^ However, it is worth noting that there are inherent limitations with diagnosing an abdominal injury after IAC-CPR. In these clinical studies, only a small percentage of the deceased patients underwent an autopsy, and in the surviving patients without abdominal symptoms, an evaluation of potential acute injury was often not undertaken. There may be increased risk of aspiration, which could be mitigated by intubation and orogastric tube placement.

This technique is by no means novel and as Sack suggested, “it is an evolution, not a revolution” in the treatment of cardiac arrest.^20^ This technique may be of particular benefit in patients who have abnormal chest wall mechanics, either through co-morbidity such as COPD or through the acquired chest wall deformity associated with prolonged compressions. Clinical consideration of the dynamic changes in chest wall movement may be beneficial during the course of resuscitation to consider modifying strategies of resuscitation. In this case one would expect the change in compliance of the chest wall would afford a more direct compression of the heart, improving systolic function; however, the poor diastolic response likely muted any benefit.

Since 2005, ILCOR has stressed improved quality and rate of chest compressions; and despite a 2003 meta-analysis demonstrating improved ROSC for IAC-CPR,^21^ there have been no large follow-up studies comparing these techniques. Ultimately the question may not be whether IAC-CPR should replace traditional CPR but rather that knowledge and practice of this technique may improve outcomes in concert with traditional methods. We need improved measures of perfusion during CPR to inform us when alternative techniques may actually improve physiological thresholds that can restore spontaneous circulation or support using external bypass devices.

Chest recoil is important regardless of the resuscitative technique. Sometimes a clinician is faced with circumstances that the literature has not addressed, such as how to resuscitate a person with no chest recoil. In those instances, having knowledge of alternative techniques may suffice to achieve the desired outcome. We feel this case illustrates a technique worth remembering.
